# Epilepsy Due to Mild TBI in Children: An Experience at a Tertiary Referral Center

**DOI:** 10.3390/jcm10235695

**Published:** 2021-12-03

**Authors:** Jun T. Park, Sarah J. DeLozier, Harry T. Chugani

**Affiliations:** 1 Epilepsy Center, UH Rainbow Babies & Children’s Hospital, Cleveland, OH 44106, USA; 2 Department of Pediatrics and Neurology, Case Western Reserve University School of Medicine, Cleveland, OH 44106, USA; 3 UH Cleveland Medical Center, Cleveland, OH 44106, USA; Sarah.Delozier@UHhospitals.org; 4 Comprehensive Epilepsy Center, NYU Langone Health, NYU School of Medicine, New York, NY 10016, USA; Harry.Chugani@nyulangone.org

**Keywords:** mild TBI, posttraumatic epilepsy (PTE), intractable epilepsy, pediatric epilepsy, TBI in children, seizures, accidental trauma, non-accidental trauma, mechanism of mild TBI, Post-impact seizures, seizure semiology

## Abstract

Rationale: Posttraumatic epilepsy (PTE) is a common cause of morbidity in children after a traumatic brain injury (TBI), occurring in 10–20% of children following severe TBI. PTE is diagnosed after two or more unprovoked seizures occurring 1-week post TBI. More often, studies have focused on children with epilepsy due to severe TBI. We aim to understand the utility of head computed tomography (HCT), EEG, and the risk of developing drug-resistant epilepsy in children after mild TBI. Method: We retrospectively studied 321 children with TBI at a tertiary pediatric referral center during a 10-year period. Mild TBI was defined as loss of consciousness (LOC) or amnesia < 30 min, moderate TBI as LOC or amnesia between 30 min and 1 day, and severe TBI as LOC or amnesia > 1 day, subdural hemorrhage, or contusion. Multiple clinical variables were reviewed, including past and present antiepileptic drug(s), seizure control, and mode of injury. First and subsequent post-TBI EEGs/prolonged video-EEGs were obtained acutely, subacutely, and/or chronically (range, day 1–3 years, median 1 month). Descriptive analyses were conducted using medians and ranges for continuous data. Categorical data were reported using frequencies and percentages, while comparisons between groups were made using Fisher’s exact test for small sample sizes. Results: Forty-seven children were diagnosed with posttraumatic epilepsy: eight children (17%) due to mild TBI, 39 children (83%) due to severe TBI. For the eight children with mild TBI whom all had an accidental trauma (non-inflicted), the median follow-up time was 25 months (range 1.5 months–84 months). The median age was 10 years (range 4–18 years), and the median age at the time of injury was seven years (range: 23 months–13 years). No relevant previous medical history was present for six patients (80%), and two patients’ (20%) relevant previous medical histories were unknown. Seven patients (88%) had no history of seizures, and patient #6 (12%) had unknown seizure history. Six patients (75%) had normal routine EEG(s). Patient #6 (13%) had an abnormal VEEG 3 months after the initial normal routine EEG, while patient #1 (13%) had an initial prolonged EEG 8 months after TBI. Compared to the 39 patients with severe TBI, 31 (79%) of whom had abnormal EEGs (routine and/or prolonged with video), mild TBI patients were more likely to have normal EEGs, *p* = 0.005. Head CT scans were obtained acutely for seven patients (90%), all of which were normal. One patient only had brain magnetic resonance imaging (MRI) 8 months after the injury. Compared to the 39 patients with severe TBI, all of whom had abnormal HCTs, mild TBI patients were less likely to have abnormal HCTs, *p* < 0.0001. In patients with mild TBI, no patient had both abnormal EEG/VEEG and HCT, and no one was on more than one Antiepileptic drug (AED), *p* < 0.005. Six patients (75%) had MRIs, of which five (63%) were normal. Two patients (#1, 7) did not have MRIs, while one patient’s (#4) MRI was unavailable. Five patients (63%) had a seizure <24 h post TBI, while the rest had seizures after the first week of injury. Conclusion: Children with epilepsy due to mild TBI, loss of consciousness, or amnesia < 30 min are more likely to have normal HCT and EEG and to be on 0–1 AED. Limitations of our study include the small sample size and retrospective design. The current findings add to the paucity of data in children who suffer from epilepsy due to mild TBI.

## 1. Introduction

According to the Centers for Disease Control in the United States, ~812,000 children visited the emergency department in 2014 after traumatic brain injury (TBI), and ~23,000 children required hospitalizations. It is estimated that most children with TBI (~90%) may fall under the category of “mild TBI” and are not even seen in emergency departments. Both severe and mild TBI’s are associated with posttraumatic epilepsy (PTE) (10–20%) [1,2,3,4,5] with a higher risk for children in the former group. PTE, diagnosed after two or more unprovoked seizures occurring 1-week post TBI, contribute significantly to the morbidity of children. Most studies on PTE have focused on children with epilepsy due to severe TBI.

The present retrospective study analyzes a cohort of children who suffered mild TBI, focusing on the utility of HCT and EEG and the likelihood of developing drug-resistant epilepsy during a 10-year period at a tertiary pediatric referral center.

## 2. Methods

### 2.1. Subject Identification

After obtaining IRB approval, a retrospective electronic chart review was performed to screen for patients who were diagnosed with TBI between 2002 and 2012 at a metropolitan tertiary pediatric referral center (Children’s Hospital of Michigan, Detroit, MI). There was a total of 321 children from inpatient and outpatient referrals to neurology service. Multiple clinical variables were collected in patients with PTE, including demographic data, relevant past medical history, history of seizure, age at TBI, type of injury, i.e., accidental (AT)/non-intentional or non-accidental (NAT)/intentional, mode of AT injury, neuroimaging and neurophysiological data, degree of TBI (mild, moderate, severe), seizure semiology, antiepileptic drug(s) (current and past), and follow-up duration. Patients were categorized into mild, moderate, or severe TBI based on clinical data. Mild TBI was strictly defined as loss of consciousness (LOC) or amnesia <30 min, while moderate TBI as LOC or amnesia between 30 min and 1 day, regardless of other clinical variables. Severe TBI was classified as LOC or amnesia >1 day, subdural hematoma, parenchymal hemorrhage, skull fracture, and/or brain contusion [6]. Post-TBI EEGs/prolonged video-EEGs were obtained acutely, subacutely, and/or chronically (range, day 1 to 3 years, median 1 month).

Researchers commonly define posttraumatic seizure (PTS) as immediate if it occurs within 24 h after TBI, early if it occurs between 24 h and 1 week after injury, and late if it occurs 1 week after TBI [7]. PTE was diagnosed if there were two or more unprovoked clinical seizures 1 week following TBI [7,8]. The diagnosis of PTE was based on available history with well-documented ictal features in follow-up neurology clinic notes. Video-EEG data with recorded seizures helped to classify seizure semiology. Epileptiform discharges on EEG, if present, supported the diagnosis of epilepsy. However, its absence did not affect the existing clinical diagnosis of PTE. Temporal information about paroxysmal epileptic events during initial hospitalization was documented well, while information about seizures that occurred weeks/months after TBI was not. If a patient had an abnormal EEG at any point, this patient was identified as having an abnormal EEG for statistical analysis. This method allowed us to be more inclusive about assigning “abnormal EEG” to a patient since not all had multiple EEGs.

### 2.2. Referral Bias

The referral practice of hospitalists and intensivists who acutely manage patients after TBI may be different. However, a child neurologist is typically consulted early on at our hospital as these patients also need outpatient follow-up. There may have been a number of patients who have been referred to an outpatient child neurologist after TBI without previous inpatient neurological consultation. However, these patients were captured in the chart review for possible diagnosis of PTE. There may also have been some patients who were not referred to a child neurologist, inpatient or outpatient, after hospitalization for TBI. These patients likely make up a very small number.

Emergency physicians identified patients who needed hospitalization. Patients who were discharged home typically are advised to contact a primary care physician for follow-up evaluation, who in turn may refer the patient to a child neurologist for symptoms such as those associated with post-concussive syndrome. All seven child neurologists in the division were board-certified and had equal participation in evaluating referred patients.

### 2.3. Inclusion and Exclusion Criteria

All patients who were seen by a child neurologist in the hospital for evaluation of seizure, focal neurological signs or symptoms, or encephalopathy after TBI were included in the chart review. In addition, new outpatient referrals for chief complaints of paroxysmal epileptic or non-epileptic event(s), focal neurological deficit, and/or post-concussive syndrome after head injury were included in the chart review. Follow-up period after a TBI ranged from nine months to eight years and five months. Only new cases of PTE, which had occurred during the 10-year period, were included.

If a patient was lost to follow-up after the first seizure, he/she was excluded. Only clinical seizures after TBI were included; therefore, patients who had subclinical seizures in intensive care units were excluded. In addition, neonates with hypoxic-ischemic injury at birth and patients with prior history of provoked or unprovoked seizures were excluded.

### 2.4. Seizure Classification and Diagnosis

Seizures were classified using the semiological classification of seizures [9] based on available history and video-EEG data. Ictal features were well documented in follow-up neurology clinic notes. Video-EEG reports also described recorded seizures in detail, allowing classification of seizures based on the degree of available information. Caretakers of patients may have missed subtle seizures and not reported them; however, this was probably insignificant given that patients with PTE had numerous seizures with clear ictal features.

### 2.5. Statistical Analysis

Descriptive analyses were conducted using medians and ranges for continuous data. Categorical data were reported using frequencies and percentages, while comparisons between groups were made using Fisher’s exact test for small sample sizes. A statically significant difference was considered present at a *p*-value < 0.05. A favorable outcome was defined by the number of antiepileptic drugs (AED) needed for seizure control.

## 3. Results

### 3.1. Characteristics of the Cohort

Overall characteristics of the entire PTE cohort had been published previously [10]. Out of 321 children with TBI, 47 (15%) children were diagnosed with PTE. Of these 47 children, 8 (17%) were associated with mild TBI (mTBI) (Table 1 and Table 2), while 39 (83%) were associated with severe TBI (sTBI). In the mTBI group, only one of the eight children was female. Head computed tomography (HCT) obtained on the day of injury was normal in seven and not obtained in one patient. None of the 39 children with sTBI had a normal HCT. A follow-up brain magnetic resonance imaging (MRI) was obtained and normal in five patients, not performed in two and unavailable in one. No child with TBI in our cohort met the criteria for moderate TBI. For the eight children with mild TBI, who all had an accidental trauma (non-inflicted), the median follow-up period was 25 months (range 1.5 months to 84 months). The median age was 10 years (range 4–18 years), and the median age at the time of injury was 7 years (range: 23 months–13 years). No relevant previous medical history was present for six patients, and two patients’ relevant previous medical histories were unknown. Seven patients had no previous history of seizures, and patient #6 had unknown seizure history. Immediate seizures occurred in five children (#1–4 and #6), who suffered either unintentional falls or unintentionally being struck by or against an object.

### 3.2. Seizure Semiology

**Figure 1:**
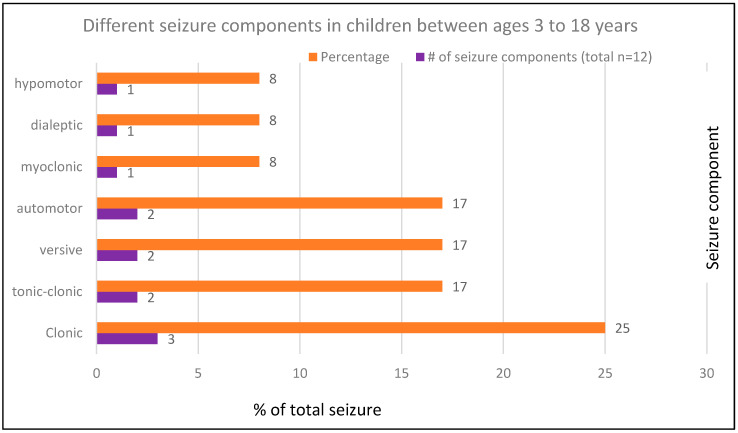
Different seizure components in children between ages 3 to 18 years.

In the mTBI group, five of the eight patients (63%) had an immediate seizure (three of these five had impact seizures), while the remaining three had seizures after the first week of injury. The most frequent seizure type was limb clonic, followed by in same frequency: versive, automotor, or tonic-clonic seizure. The least frequent seizure types were hypomotor, dialeptic, or myoclonic seizures. No patient who had mTBI had epileptic spasms (Figure 1). Details of epileptic spasms in posttraumatic epilepsy from our cohort have been published previously [11].

### 3.3. Antiepileptic Drug

Two (#1 and #8) of the eight patients with mTBI had been on PHT and PHB in the past, respectively. Six patients were on one AED: four on levetiracetam and two on valproic acid. Patient #8 was weaned off valproate due to pancytopenia and had not been started on a new AED at the time of data collection. In comparison, of the 39 children who had suffered severe TBI, 19 patients (49%) were on 2 or more AEDs, and 15 (38%) were on one AED [10].

### 3.4. Electroencephalograph

Seven of the eight patients with mTBI had an initial normal routine EEG. Patient #6 had an abnormal VEEG showing epileptiform discharges, including polyspikes, in the left hemisphere 1 month after the initial normal routine EEG (Table 1 and Table 2). Patient #1 had his first EEG 8 months after TBI, which showed 3 Hz spike-and-slow waves with bi-frontal predominance (Table 1 and Table 2). Compared to the 39 patients with severe TBI, 31 (79%) of whom had abnormal EEGs (routine and/or prolonged with video), mTBI patients were more likely to have normal EEGs, *p* = 0.005 (Table 3). In patients with mTBI, no one had both abnormal EEG/VEEG and head computed tomography (Table 4), and no one was taking more than one AED, *p* < 0.005 (Table 5).

### 3.5. Head Imaging

Head CT scans were obtained acutely for seven patients (90%), all of which were normal. One patient only had brain magnetic resonance imaging (MRI) 8 months after the injury. Compared to the 39 patients with severe TBI, all of whom had abnormal HCTs, mild TBI patients were less likely to have abnormal HCTs, *p* < 0.0001 (Table 6). Six patients (75%) had MRIs, of which five (63%) were normal. Two patients (#1, 7) did not have MRIs, while one patient’s (#4) MRI was unavailable. Five patients (63%) had a seizure <24 h post TBI, while the rest had seizures after the first week of injury.

### 3.6. Mechanism of Injury

**Figure 2:**
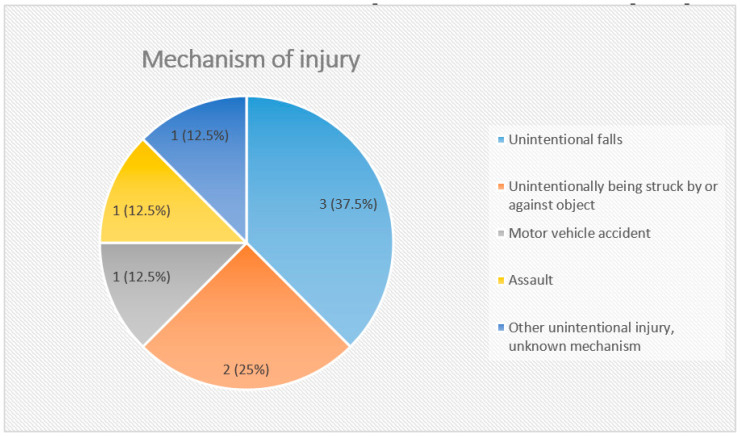
Pie chart showing distribution of children (total *n* = 8) by mechanism of injury.

One (#5) of the eight patients with mTBI was struck with a rock by a classmate, while the remaining cohort had accidental injuries. The mode of injury for seven children involved an impact with a moving object (rock, head, or car) or a stationary entity (concrete floor, ice floor, wall, or stairs), while patient #8 had a “closed head injury”. More specifically, the mechanism of injury was falling (3 children), being struck by or against an object (2), motor vehicle accident (1), and assault (1) (Figure 2). Immediately following TBI, only patient #4 had a loss of consciousness, while this information was unavailable for patient #8. The loss of consciousness in patient #4 was immediate and not part of the postictal state.

## 4. Discussion

### 4.1. Mechanism of Injury

The Center for Disease Control in the United States found that from 2006 to 2014, the three most common mechanisms of TBI in adults and children who visited emergency departments were unintentional falls (47.9%), followed by unintentionally being struck by or against an object (17.1%), and then followed by motor vehicle accidents (13.2%) [12]. Our cohort of eight children who had mTBI also showed the same trend in the frequency of injuries by these three mechanisms (Figure 2).

### 4.2. “Mild” TBI

Frequently, investigators in the field of PTE have used the Glasgow Coma Scale of 1315 to classify mTBI [13,14,15], while some have used clinical variable(s) to define it [6,11,16,17]. Since there is no precise definition of mild TBI, it has been used interchangeably with a concussion in the literature [18,19]. In addition, since most patients with concussion/mTBI do not present to the hospital, studies that only include patients in the hospital registry very likely underreport the number of patients with mTBI [20,21,22]. Another confound that may contribute to inaccurate estimation of risk of PTE after mTBI is that there are patients with generalized or focal seizure tendency before they experience their first seizure after head injury [23]. The cause of PTE in this group of children is probably due to underlying seizure tendency and not necessarily due to TBI. In our cohort, patient #1 with no prior history of seizure probably had genetically influenced seizure tendency before TBI. EEG 8 months after injury (first EEG) showed generalized 3 Hz spike-and-slow waves, a pattern that is unlikely to be the consequence of TBI. Patient #6 had an uncomplicated birth, no prior seizure history, and normal head CT and brain MRI. However, she had unexplained global developmental delay, hypotonia, and visual impairment preceding TBI. Her routine EEG (20 min in duration) 2 months after injury was normal; however, 3 months post TBI, a 24-h video EEG showed left-hemispheric spikes in NREM.

### 4.3. Impact Seizure

Based on video analysis of adult athletes, impact seizures have been described to occur within 2 s of head injury and are characterized by loss of consciousness and a brief tonic phase followed by myoclonic or clonic activity of the extremities, lasting less than 150 s [24,25]. These studies have shown that impact seizures do not increase the risk of PTE in adults after sport-related TBI. The loss of consciousness as a defining feature at the time of impact seizure, however, may not be applicable to children. In our cohort, two patients experienced impact seizures without loss of consciousness. Patient #3 (9-year-old boy) experienced an altered sensorium after hitting his head on the ice while skating and subsequently had a seizure. Patient #6 (23-month-old girl) fell down two or three steps in a stairway and had an impact seizure, after which she cried immediately. A seizure occurred 30 min after the impact in patient #2 (6-year-old boy) after hitting his head on concrete. The precise duration of these seizures is unavailable, although thought to be “brief.” These cases suggest that (1) loss of consciousness may not occur at the time of an impact in young children who have impact seizures; (2) children who have impact seizures may develop PTE. Further, a subcategory of “Post-impact seizures”, seizures that occur 150 s after and probably within an hour of an impact may need further investigation in all age groups. It is unknown whether there is an association between the degree of impact at the time of TBI and the risk of impact seizure with or without loss of consciousness in children.

### 4.4. Seizure Semiology

The frequency of seizure types in this cohort (Figure 1) with a median age of 10 years (range 4–18 years) aligns with the findings by Fernandez Baca-Vaca et al. (2018) [26], who reviewed and classified video-EEG recordings of seizures in 270 children and adults. These investigators found that seizure types differ by age groups, and in children aged 6–18 years, focal tonic or clonic seizure was the most frequent type, followed by versive, automotor, and generalized tonic-clonic seizure. It is well known that seizure semiology in children becomes more elaborate as the brain matures [26].

### 4.5. Diagnostic Studies in Mild TBI

Many diagnostic studies were normal in our mTBI cohort. Head CT was normal in seven patients (not performed in 1), while brain MRI was normal in five (one unavailable, two not performed). Standard outpatient EEG obtained 1 day to 6 weeks after TBI was also normal in all seven patients (not performed in 1). All children had well-controlled epilepsy while taking one or no AED. For six of the eight children, seizure recurrence (not EEG results) contributed to the diagnosis of epilepsy and the decision to initiate AED therapy. The remaining two children (patient #1, #6) likely had seizure tendencies preceding TBI. It is unlikely that spot EEG post TBI (mild or severe) predicts the risk of PTE in children. Investigators in the 1960s and 1970s had corroborated this. Jennett and van de Sande (1975) [27] from two different centers investigated serial EEGs of 772 adult patients post TBI and found that “although abnormal records were more common in patients who developed epilepsy, they reflected the more severe brain damage in these patients, which was already evident from clinical features.” They concluded that in “individual patients the EEG does not improve the accuracy of the prediction calculated from clinical data.” Their finding comported with those of Walton (1963) [28], Courjon (1969) [29], and Terespolsky (1972) [30].

In contrast, a recent study of 50 hospitalized adults whose EEG showed epileptiform discharges within 5 days of TBI reported that epileptiform abnormalities post TBI could identify patients at risk of PTE [31]. The authors analyzed data only up to 12 months post TBI. Twenty-five patients who developed PTE were compared to 25 controls without PTE, matched by age and admission Glasgow Coma Score (GCS) (6 ± 4.66 (PTE group) and 8 ± 4.53 (No PTE group)). All 50 patients with a median age of 52.5 ± 20.4 (PTE group) and 49.6 ± 25.8 (No PTE group) had imaging abnormalities, including intraparenchymal hemorrhage, subdural hemorrhage, subarachnoid hemorrhage, and/or epidural hemorrhage. Their conclusion may not apply to children and adults with mild TBI. The study represented hospitalized adults with likely moderate to severe TBI, reflected by the GCS and neuroimaging abnormalities. Their finding may be explained by the fact that the increased frequency of abnormal EEG, more commonly seen in patients who develop PTE, reflects the more severe brain injury already apparent from clinical and neuroimaging data, as concluded by the aforementioned researchers [27,29,30].

### 4.6. Risk of PTE

The risk of PTE is associated with the mechanism and severity of injury [6]. Posttraumatic seizures are more frequent in children after inflicted versus accidental TBI. [10,11,32,33,34]. Only one of our eight patients with mTBI was intentionally struck with a rock by another child. A recent study examined the records of 2122 children (<18 years of age) with severe TBI (sTBI) (Glasgow Coma Scale score ≤ 8 and hospital length of stay more than 24 h) in the National Trauma Databank and Pediatric Health Information Systems. PTE occurred in 57% of children aged less than 2 years with subdural hemorrhage [35]. In our previous studies, patients with sTBI also had an increased risk of having multiple seizure types, being on two or more AEDs to control seizures, and were at risk of developing epileptic spasms [10,11].

The risk of epilepsy in children after mTBI is probably higher than that of the general population at 0.7% [15,36], but lower than the risk due to sTBI (~10–20%) [3,4,5,10]. In our cohort, 47 (15%) out of 321 children who suffered TBI developed PTE, which is in the reported range of previous studies, including a population-based study [16,36]. A total of 8 (17%) of the 47 children had seizures post mTBI, suggesting increased risk in this particular group. However, they are more likely to have normal EEGs, *p* = 0.005 (Table 3) and seizure control on 0–1 AED, *p* < 0.005 (Table 5). The total number of children with mTBI who did not develop seizures is unknown as we only evaluated those children who developed PTE and were seen in the hospital or clinic by a child neurologist.

### 4.7. Study Limitations

Due to the retrospective nature of the present study, details of clinical variables such as duration of impact seizures and the precise onset of immediate and late seizures were limited to the extent of documentation in charts. As alluded to above, the number of children with posttraumatic seizures may have been underrepresented because those who had only one seizure at the time of chart review were excluded. Some of these children may have had a subsequent second unprovoked seizure. Given the small sample size of children with mTBI, our findings involving impact seizures as defined in our study need further validation in a larger cohort.

### 4.8. Future Direction

First, a common definition of mTBI needs to be explored for research and clinical use. This would allow the comparison of data from different research studies, as well as estimation of more precise risk of PTE and outcome in different age groups. Using clinical variables only to define mTBI may help avoid unnecessary radiation from head CT and sedation to obtain brain MRI. Indeed, the CDC Pediatric mTBI Guideline recommends: (1) Do not routinely image patients to diagnose mTBI. (2) Use validated, age-appropriate symptom scales to diagnose mTBI [12]. Impact seizures in children need further investigation and characterization as impact seizures in professional adult athletes may not be synonymous with those in children.

## 5. Conclusions

Children who suffer mild TBI, loss of consciousness, or amnesia <30 min are more likely to have normal HCT and EEG (acute or chronic) and to be on 0–1 AED. They are probably at higher risk for epilepsy relative to the general population. The current findings add to the accumulating data in children who suffer from epilepsy due to mild TBI.

## Figures and Tables

**Table 1:** Eight of 47 children who diagnosed with mild TBI.

Patient #	Age	Sex/Age (Months or Year) @ Injury	Relevant PMH	History of SZ	Type of Injury: AT/NAT	Post-TBI EEG (Time)/Result	Post TBI Prolonged EEG (Time)/Duration/Result	CT	MRI Pathology	Mechanism of Mild TBI/Direction of Acceleration
1	11y	M/8y	none	no	AT	Not done	8 months/1 day/discharges of 3 Hz spike-and-slow waves with bifrontal predominance	nmL	not done	Head vs. head: Hit his head against another child’s head/Horizonal
2	7y	M/6y	none	no	AT	1 day/nmL 3 months/nmL	-	nmL	nmL	Head vs. concrete: Hit his head on concrete/Vertical
3	10y	M/9y	none	no	AT	1 day/nmL	-	nmL	nmL	Head vs. ice: Hit left side of his head while skating/Vertical
4	4y	M/3y	none	no	AT	6 weeks/nmL	-	not done	unavailable	Head vs. concrete: Forehead into a wall while running/Horizonal
5	18y	M/8y	none	no	AT	2 years/nmL 3 years/nmL	2 yrs/1 day/normal	nmL	nmL	Head vs. rock: Attacked by a classmate with a rock, striking him in the posterior head region/Horizonal
6	4y	F/23 mo	uncomplicated birth, GDD, hypotonia and visual impairment	unknown	AT	2 months/nmL	3 months/1 day/spike and slow waves, polyspikes in the left central-temporal-parietal region in NREM	nmL	nmL	Head vs. stairs: Fell down two or three stairs/Vertical
7	18y	M/11y	unknown	no	AT	1 month/nmL	8 years/1 day/normal	nmL	not done	Scooter vs. car: He was on a scooter when hit by a car/Horizonal & Vertical
8	5 y	M/4y	unknown	no	AT	1 month/nmL	-	nmL	nmL	Unknown: “closed head injury”/Unknown

**Table 2:** Eight of 47 children who diagnosed with mild TBI-Continued from Table 1.

Patient #	Age	Sex/Age (Months or Year) @ Injury	LOC	Impact SZ	SZ < 1 h of TBI	SZ < 24 h of TBI	>1 d to <1 wk (Number of Seizure)	>8 d	Semiology Classification	Past AED	Current AED	Follow Up Duration (Months)	Diagnosis of Epilepsy at Data Collection
1	11y	M/8y	no		-	yes	-	yes	generalized clonic seizure	PHT	LEV	54 mo	yes
2	7y	M/6y	no		Yes	-	-	yes	right versive → dialeptic seizure	none	LEV	7 mo	yes
3	10y	M/9y	no	Yes	-	-	-	yes	automotor → simple motor seizure	none	LEV	14 mo	yes
4	4y	M/3y	yes	Yes	-	-	-	yes	right leg clonic seizure	none	none	1.5 mo	yes
5	18y	M/8y	no	-	-	-	-	yes	1. hypomotor seizure 2. tonic-clonic seizure	none	VPA	80 mo	yes
6	4y	F/23 mo	no	yes	-	-	-	yes	1. leftt versive seizure 2. tonic-clonic seizure	none	LEV	38 mo	yes
7	18y	M/11y	no	-	-	-	-	yes	automotor seizure	none	VPA	84 mo	yes
8	5 y	M/4y	-	-		-	-	yes	right face and leg clonic/myoclonic seizure	PHB	VPA -off (pancyto-penia)	2 mo	yes

**Table 3:** Abnormal vs. normal EEG and/or VEEG (acute and chronic).

EEG and/or VEEG	Severe TBI	Mild TBI	*p*-Value
Abnormal	31	2	*p* = 0.005
Normal	8	6	

TBI: traumatic brain injury.

**Table 4:** Comparison of severe vs. mild TBI patients: Number of patients with abnormal EEG/VEEG only vs. abnormal EEG/VEEG + abnormal HCT, and number of AED(s) for each group.

		Severe TBI: *n* = 39			Mild TBI: *n* = 8	
	Number of Patients	≥2 AEDs (*n* = 19)	0–1 AED (*n* = 20)	Number of Patient/Total *n*	≥2 AEDs (*n* = 0)	0–1 AED (*n* = 10)
Abnormal EEG/VEEG	38	-	-	2	-	-
Unavailable EEG/VEEG	1	-	-	0	-	-
Abnormal EEG/VEEG only	0	-	-	2/8 (25%)	0	2/8 (25%)
Both abnormal EEG/VEEG and HCT	29 (74%)/39	14 (48%)	15 (52%)	0/8	0	0

**Table 5:** Abnormal EEG and/or VEEG + number of AEDs among patients with mild TBI.

Number of AEDs	Observed Frequency	Expected Frequency under the Null Hypothesis	*p*-Value
2 + AEDs	0 (0%)	4 (50%)	
0–1 AEDs	8 (100%)	4 (50%)	*p* = 0.005

**Table 6:** Abnormal vs. head computed tomography (CT).

Head CT	Severe TBI	Mild TBI	*p*-Value
Abnormal	39	0	*p* < 0.0001
Normal	0	7	

## Data Availability

Not applicable.

## References

[1] Ong L., Dhillon M., Selladurai B., Maimunah A., Lye M. (1996). Early post-traumatic seizures in children: Clinical and radiological aspects of injury. J. Paediatr. Child Health.

[2] Hahn Y.S., Fuchs S., Flannery A.M., Bartehl M.J., McLone D.G. (1988). Factors influencing posttraumatic seizures in children. Neurosurgery.

[3] Ratan S.K., Kulshreshtha R., Padey R.M. (1999). Predictors of posttraumatic convulsions in head injured children. Pediatr. Neurosurg..

[4] Barlow K.M., Spowart J.J., Minns P.A. (2000). Early posttraumatic seizures and nonaccidental head injury: Relation to outcome. Dev. Med. Child Neurol..

[5] Appleton R.E., Demellweek C. (2002). Post-traumatic epilepsy in children requiring inpatient rehabilitation following head injury. J. Neurol. Neuosurg. Psychiatry.

[6] Annegers J.F., Coan S.P. (2000). The risks of epilepsy after traumatic brain injury. Seizure.

[7] Annegers J.F., Grabow J.D., Groover R.V., Laws E.R., Elveback L.R., Kurland L.T. (1980). Seizures after head trauma: A population study. Neurology.

[8] Adelson P.D., Bratton S.L., Carney N.A., Chesnut R.M., du Courday H.E.M., Goldstein B., Kochanek P.M., Miller H.C., Partington M.D., Selden N.R. (2003). Guidelines for the acute medical management of severe traumatic brain injury in infants, children, and adolescents. The role of anti-seizure prophylaxis following severe pediatric traumatic brain injury. Pediatr. Crit. Care Med..

[9] Luders H., Acharya J., Baumgartner C., Benbadis S., Bleasel A., Burgess R., Dinner D.S., Ebner A., Foldvary N., Geller E. (1998). Semiological Seizure Classification. Epilepsia.

[10] Park J.T., Chugani H.T. (2015). Post-traumatic epilepsy in children-Experience from a tertiary referral center. Ped. Neurol..

[11] Park J.T., Chugani H.T. (2017). Epileptic spasms in paediatric post-traumatic epilepsy at a tertiary referral centre. Epileptic Disord..

[12] Centers for Disease Control. https://www.cdc.gov/traumaticbraininjury/data/tbi-edhd.html.

[13] Mena J.H., Sanchez A.I., Rubiano A.M., Peitzman A.B., Sperry J.L., Gutierrez M.I., Puyana J.C. (2011). Effect of the Modified Glasgow Coma Scale Score Criteria for Mild Traumatic Brain Injury on Mortality Prediction: Comparing Classic and Modified Glasgow Coma Scale Score Model Scores of 13. J. Trauma Inj. Infect. Crit. Care.

[14] Huguenard A.L., Miller B.A., Sarda S., Capasse M., Reisner A., Chern J.J. (2016). Mild traumatic brain injury in children is associated with a low risk for posttraumatic seizures. J. Neurosurg. Pediatr..

[15] Keret A., Bennett-Back O., Rosenthal G., Gilboa T., Shweiki M., Shoshan Y., Benifla M. (2017). Posttraumatic epilepsy: Long-term follow-up of children with mild traumatic brain injury. J. Neurosurg. Pediatr..

[16] Annegers J.F., Hauser W.A., Coan S.P., Rocca W.A. (1998). A Population-Based Study of Seizures after Traumatic Brain Injuries. N. Engl. J. Med..

[17] Gilad R., Boaz M., Sadeh M., Eilam A., Dabby R., Lampl Y. (2013). Seizures after Very Mild Head or Spine Trauma. J. Neurotrauma.

[18] McCrory P., Meeuwisse W.H., Aubry M., Cantu B., Dvořák J., Echemendia R.J., Engebretsen L., Johnston K., Kutcher J.S., Raftery M. (2013). Consensus statement on concussion in sport: The 4th International Conference on Concussion in Sport held in Zurich, November 2012. Br. J. Sports Med..

[19] McCrory P., Meeuwisse W., Dvorak J., Aubry M., Bailes J., Broglio S., Cantu R.C., Cassidy D., Echemendia R.J., Ruben J. (2017). Consensus statement on concussion in sport—the 5th international conference on concussion in sport held in Berlin, October 2016. Br. J. Sports Med..

[20] Mahler B., Carlsson S., Andersson T., Adelo W.C., Ahlbom A., Tomson T. (2015). Unprovoked seizures after traumatic brain injury: A population based case-control study. Epilepsia.

[21] Christensen J., Pedersen M.G., Pedersen C.B., Sidenius P., Olsen J., Vestergaard M. (2009). Long-term risk of epilepsy after traumatic brain injury in children and young adults: A population-based cohort study. Lancet.

[22] Nguyen R., Fiest K.M., McChesney J., Kwon C.-S., Jette N., Frolkis A.D., Atta C., Mah S., Dhaliwal H., Reid A. (2016). The International incidence of traumatic brain injury: A systemic review and meta-analysis. Can. J. Neurol. Sci..

[23] Tai P., Gross D. (2004). Exacerbation of Pre-existing Epilepsy by Mild Head Injury: A Five Patient Series. Can. J. Neurol. Sci..

[24] McCrory P.R., Bladin P.F., Berkovic S. (1997). Retrospective study of concussive convulsions in elite Australian rules and rugby league footballers: Phenomenology, aetiology, and outcome. BMJ.

[25] McCrory P.R., Berkovic S.F. (1998). Coincussive convusions. Incidence in sport and treatment recommendations. Sprots Med..

[26] Fernandez G., Mayor C., Losarcos N., Park J., Luders H. (2018). Epileptic seizure semiology in different age groups. Epileptic Disord..

[27] Jennett B., Van De Sande J. (1975). EEG Prediction of Post-Traumatic Epilepsy. Epilepsia.

[28] Walton J.N. (1963). Some observations on the value of electroencephalography in medicolegal practice. Med. Leg. J..

[29] Terespolsky P.S. (1972). Post-traumatic epilepsy. Forensic Sci..

[30] Kim J.A., Boyle E.J., Wu A.C., Cole A.J., Staley K.J., Zafar S., Cash S.S., Westover M.B. (2018). Epileptiform activity in traumatic brain injury predicts post-traumatic epilepsy. Ann. Neurol..

[31] Keenan H.T., Runyan D., Marshall S., Nocera M.A., Merten D.F. (2004). A Population-Based Comparison of Clinical and Outcome Characteristics of Young Children with Serious Inflicted and Noninflicted Traumatic Brain Injury. Pediatrics.

[32] Vinchon M., Defoort-Dhellemmes S., Desurmont M., Dhellemmes P. (2005). Accidental and non-accidental head injuries in infants: A prospective study. J. Neurosurg..

[33] Ewing-Cobbs L., Kramer L., Prasad M., Canales D.N., Louis P.T., Fletcher J.M., Vollero H., Landry S.H., Cheung K. (1998). Neuroimaging, Physical, and Developmental Findings After Inflicted and Noninflicted Traumatic Brain Injury in Young Children. Pediatrics.

[34] Bennett K.S., DeWitt P., Harlaar N., Bennett T. (2017). Seizures in Children with Severe Traumatic Brain Injury. Pediatr. Crit. Care Med..

[35] Temkin D.R. (2003). Risk factors for posttraumatic seizures in adults. Epilepsia.

[36] Hauser W.A., Annegers J.F., Kurland L.T. (1993). Incidence of Epilepsy and Unprovoked Seizures in Rochester, Minnesota: 1935-1984. Epilepsia.

